# Optic Disc Morphology and Paracentral Scotoma in Patients with Open-Angle Glaucoma and Myopia

**DOI:** 10.3390/jcm12093295

**Published:** 2023-05-05

**Authors:** Minha Kim, Euntak Hong, Eun Ji Lee

**Affiliations:** 1 Seoul National University College of Medicine, Seoul 03080, Republic of Korea; 2 Department of Ophthalmology, Seoul National University Bundang Hospital, Seongnam 13620, Republic of Korea

**Keywords:** open-angle glaucoma, myopia, optic disc tilt, paracentral scotoma, peripheral scotoma

## Abstract

This study’s aim was to investigate the association between optic disc morphology and the occurrence of paracentral scotoma in eyes with open-angle glaucoma (OAG) and myopia. Two-hundred and eleven myopic eyes with OAG were classified into three groups according to the location of visual field (VF) defect (99 paracentral scotoma, 65 peripheral scotoma, and 47 no VF defect). Optic disc morphology was assessed based on the tilt ratio and eccentricity of the central retinal vessel trunk (CRVT). Clinical characteristics of the three groups were compared, and factors affecting the occurrence of paracentral scotoma were determined. Eyes with paracentral scotoma had a higher tilt ratio than the other groups (*p*s ≤ 0.04). Multiple linear regression showed that a nasal location of CRVT (*p* < 0.001), longer axial length (*p* = 0.001), and lower VF mean deviation (*p* = 0.021) were significantly associated with higher tilt ratio. In logistic regression analysis, tilt ratio was the only factor that was significantly associated with the occurrence of paracentral scotoma (odds ratio = 7.12, *p* = 0.032). In conclusion, the optic disc tilt ratio increased with nasal shift of CRVT, longer axial length, and lower VF mean deviation. Higher optic disc tilt was significantly associated with the occurrence of paracentral scotoma in eyes with OAG and myopia.

## 1. Introduction

Myopia is a well-established risk factor for glaucoma [1,2,3,4]. Although the underlying pathogenic mechanisms remain unclear, morphologic changes in the optic nerve head (ONH) during the development of myopia may be associated with the pathogenesis of glaucoma in myopic eyes [5,6,7,8]. Axial elongation associated with the progression of myopia induces peripapillary scleral deformation, along with changes in the ONH, such as an oval optic disc, frequently called an optic disc tilt. This process may exert mechanical stresses on peripapillary and ONH tissues, damaging axons in the ONH [9,10,11,12,13,14]. The risk of glaucoma has been reported to be higher in eyes with than without tilted optic discs, with the degree of tilt being positively associated with the severity of visual field (VF) damage [8,12,15,16,17].

VF defects near fixation are the most significant consequences of glaucoma, as they can markedly reduce patient quality of life [18]. Highly myopic eyes with optic disc tilt were reported to be prone to developing paracentral VF defects. Papillomacular bundles, which are responsible for paracentral VF, are more frequently damaged in highly myopic eyes, even in the early stages of glaucoma [12]. Moreover, high myopia and optic disc tilt were found to be risk factors for papillomacular bundle damage. 

Previous studies assessing the relationship between optic disc tilt and the occurrence of paracentral scotoma have yielded conflicting results [19,20,21]. These studies were not directly comparable, however, as the patients enrolled differed in the degree of myopia and/or the types and severity of glaucoma, indicating a need for further research. 

The present study was designed to investigate the association of optic disc tilt with paracentral scotoma in Korean patients with myopia and open-angle glaucoma (OAG). Clinical characteristics associated with the degree of optic disc tilt were determined, as were factors affecting the occurrence of paracentral scotoma.

## 2. Materials and Methods

### 2.1. Subjects

This study included patients with OAG and myopia who were enrolled in the Investigating Glaucoma Progression Study, an ongoing prospective study of glaucoma patients at the Glaucoma Clinic of Seoul National University Bundang Hospital. Medical records obtained between January 2016 and November 2022 were reviewed. The study protocol was approved by the institutional review board of Seoul National University Bundang Hospital and followed the tenets of the Declaration of Helsinki.

All subjects underwent comprehensive ophthalmic examinations, including assessment of best-corrected visual acuity, refraction tests, slit-lamp biomicroscopy, gonioscopy, Goldmann applanation tonometry, stereo disc photography, red-free fundus photography (EOS D60 digital camera, Canon, Utsunomiyashi, Japan), and standard automated perimetry (Humphrey Field Analyzer II 750, 24-2 Swedish interactive threshold algorithm, Carl Zeiss Meditec, Dublin, CA, USA). Circumpapillary RNFL thickness was measured and the ONH scanned using spectral domain OCT (Spectralis, Heidelberg Engineering, Heidelberg, Germany). Other ophthalmic examinations included measurements of corneal curvature (KR-1800, Topcon, Tokyo, Japan), central corneal thickness (CCT; Orbscan II, Bausch & Lomb Surgical, Rochester, NY, USA), and axial length (AXL; IOL Master version 5, Carl Zeiss Meditec).

OAG was defined as an open angle on gonioscopy, and signs of glaucomatous optic nerve damage (e.g., neuroretinal rim notching, thinning, or a localized retinal nerve fiber layer (RNFL) defect). A glaucomatous VF defect was defined as a defect conforming to one or more of the following criteria: (1) results outside normal limits on glaucoma hemifield tests, (2) at least three adjacent abnormal points with a *p* < 0.05 probability of being normal and at least one with a *p* < 0.01 probability by pattern deviation, or (3) a pattern standard deviation of *p* < 0.05, confirmed on two consecutive reliable tests. The OAG eyes without glaucomatous VF defect were considered as preperimetric glaucoma and were included in the “no VF defects” group (see, Section 2.3).

Eyes were included if they were myopic, with a spherical refraction ≤ −0.75 diopters (D) or AXL > 24.0 mm. Eyes that underwent cataract extraction had to conform to the AXL criterion. Eyes were excluded if they had a best-corrected visual acuity (BCVA) below 20/40, cylinder correction less than −3.0 diopters or more than +3.0 diopters, pathologic myopia, a history of previous intraocular surgery other than uneventful cataract surgery or trabeculectomy, or retinal or neurologic diseases that could produce VF defects. Eyes were also excluded if the image quality was too poor to allow the proper evaluation of optic disc morphology. If both eyes of a subject were eligible, one was randomly selected for inclusion in the study.

### 2.2. Determination of Optic Disc Morphology

**Figure 1:**
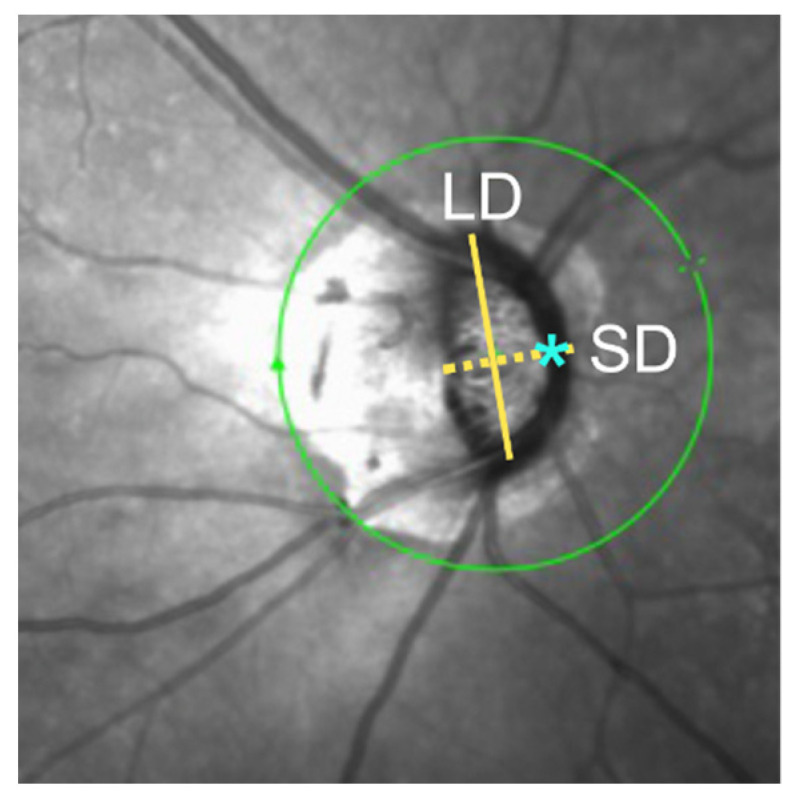
Determination of optic disc tilt ratio and the location of CRVT. Tilt ratio was calculated as the ratio between the longest diameter (LD; solid yellow line) and the shortest diameter (SD; dashed yellow line) of the optic disc (LD/SD). CVRT location (asterisk) was based on the location of exit of the CRVT on the lamina cribrosa surface relative to the temporal disc border at the short disc axis. The distance between the CRVT and the temporal disc border divided by the short disc diameter was calculated. Ratios between 0.3 and 0.7 were considered central and ratios > 0.7 were considered nasal.

Optic disc morphology was assessed based on the eccentricity of the central retinal vessel trunk (CRVT) and optic disc tilt ratio on OCT infrared images (Figure 1). Optic disc tilt ratio was defined as the ratio of the longest diameter (LD) to the shortest diameter (SD) of the optic disc [8]. Two independent examiners measured the LD and SD, and the mean of the two measurements was used in the final analysis. 

CRVT location was based on the location of the exit of the CRVT on the lamina cribrosa surface relative to the temporal disc border at the short disc axis. Eyes were categorized into two groups: those with nasal and central CRVT. The distance between the CRVT and the temporal disc border was divided by the short disc diameter [22], with ratios between 0.3 and 0.7 considered central and ratios > 0.7 considered nasal.

### 2.3. Determination of the Location of Visual Field Defect

**Figure 2:**
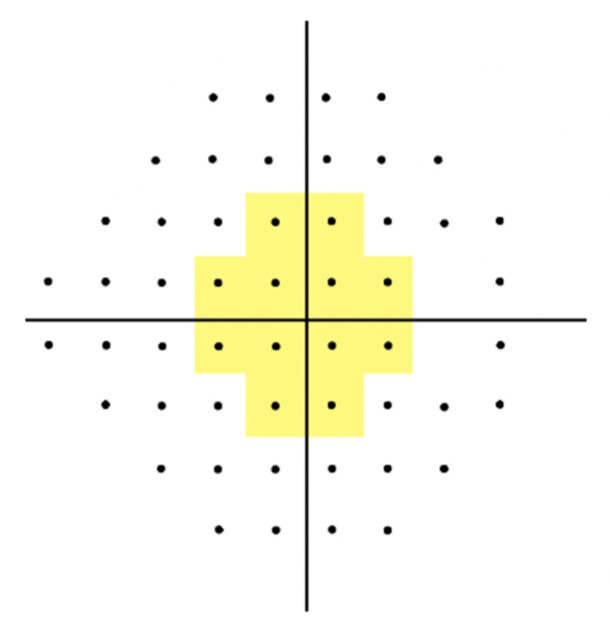
Pattern deviation plot dividing locations of paracentral and peripheral scotomas. Abnormal points within 12 points of a central 10° radius (yellow area) and those outside this area were defined as paracentral and peripheral scotomas, respectively.

VF defect locations were categorized as central or peripheral based on the pattern deviation probability plots in 24-2 Swedish interactive threshold algorithm tests. Abnormal points within 12 points of a central 10° radius were defined as paracentral scotoma, whereas those in sectors outside the central 10° radius were defined as peripheral scotoma (Figure 2). Eyes were classified into three groups according to the location of the VF defect: those with paracentral scotoma, those without paracentral scotoma but with peripheral scotoma, and those without VF defects. Eyes with both paracentral and peripheral scotomas were classified into the paracentral scotoma group, while those having only peripheral scotoma but no VF defects within the central 10° radius were classified into the peripheral scotoma group.

### 2.4. Statistical Analysis 

Interobserver agreement in the determination of the CRVT location was assessed using Cohen’s kappa statistics. Interobserver variability in the optic disc tilt ratio was assessed by measuring the intraclass correlation coefficient (ICC) and its confidence interval (CI), and 95% Bland–Altman limits of agreement. Continuous variables were compared using one-way analysis of variance (ANOVA) with Tukey’s post hoc test, and categorical variables were compared using chi-square tests. A general linear model was used to identify the factors affecting the optic disc tilt ratio. Factors associated with the occurrence of paracentral scotoma were determined through logistic regression analysis. Variables with *p*-values < 0.10 in the univariable model were included in the multivariable model. All statistical analyses were performed using SPSS version 19.0 (SPSS Inc., Chicago, IL, USA), with *p*-values < 0.05 considered statistically significant. 

## 3. Results

Of the 211 eyes with OAG and myopia included in this study, 99 eyes had paracentral scotoma, 65 had peripheral scotoma, and 47 had no VF defects. Cohen’s kappa coefficient for the determination of CRVT location was 0.95. The ICC for the measurement of the tilt ratio was 0.930 (95% confidence interval (CI), 0.913–0.943), and the 95% Bland–Altman limits of agreement were −0.281 and 0.270. Table 1 summarizes and compares the demographic and clinical characteristics of the three groups. There were no statistically significant differences in age, sex, location of CRVT, or baseline IOP, SEQ, AXL, and CCT among these groups (all *p* > 0.10). The optic disc tilt ratio was significantly higher in eyes with paracentral scotoma than in the other groups (*p* = 0.018), but was comparable in eyes with peripheral scotoma and those without VF defects (*p* = 0.627). The visual field MD (*p* = 0.227) and PSD (*p* = 0.481) did not differ significantly between eyes with paracentral and peripheral scotoma. The global RNFLT and visual field MD were larger, whereas the visual field PSD was smaller, in eyes without VF defects than in the other two groups (all *p* < 0.001).

Because the optic disc tilt ratio was the only factor that differed significantly between eyes with paracentral and peripheral scotoma, factors associated with the optic disc tilt ratio were determined using a general linear model (Table 2). Univariable analysis showed that a nasal location of CRVT (*p* < 0.001), larger AXL (*p* = 0.001), and worse visual field MD (*p* = 0.022) were significantly associated with a higher tilt ratio. Multivariable analysis that included all factors with *p* < 0.1 in univariable analyses showed that nasal CRVT location (*p* < 0.001), larger AXL (*p* = 0.001), and worse visual field MD (*p* = 0.021) were independent predictors of a higher optic disk tilt ratio. 

Table 3 shows the results of a logistic regression analysis assessing the factors associated with the occurrence of paracentral scotoma in eyes with paracentral and peripheral scotoma. A higher tilt ratio was the only factor that was significantly associated with the occurrence of paracentral scotoma in univariable analysis (odds ratio (OR) = 4.498, 95% CI = 1.043–19.397; *p* = 0.044). Multivariable analysis was performed including the variables that were significantly associated with the optic disc tilt ratio (Table 2), together with the tilt ratio, to account for potential confounding effects of the variables. Additionally, in the multivariable analysis, only the optic disc tilt ratio was a significant factor for the presence of paracentral scotoma (odds ratio (OR) = 8.832, 95% CI = 1.369–56.960; *p* = 0.022).

**Figure 3:**
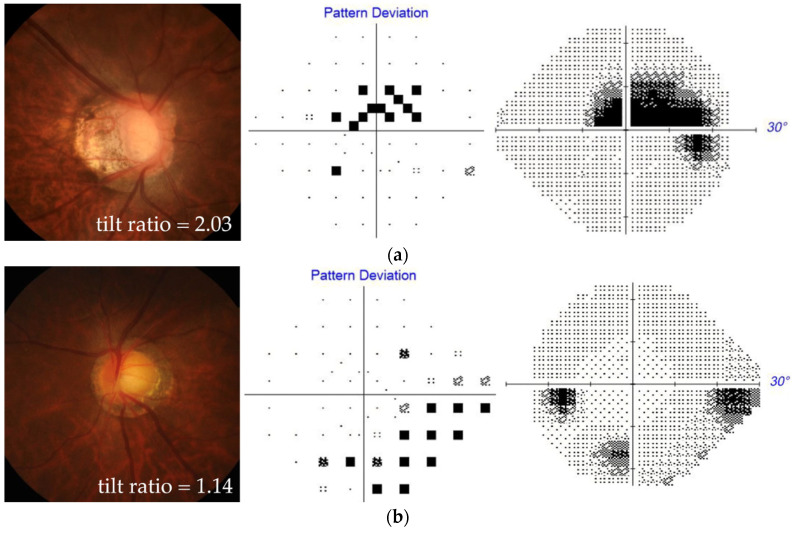
Two representative patients with myopia and OAG with different degrees of optic disc tilt, one having paracentral scotoma (**a**), and the other having peripheral scotoma (**b**). For each patient, stereo disc photography (**left**) and perimetry results in a pattern deviation plot (**middle**) and grayscale map (**right**) are presented.

Figure 3 shows two representative patients with myopia and OAG with different degrees of optic disc tilt, one having paracentral (Figure 3a) and the other having peripheral (Figure 3b) VF defects.

## 4. Discussion

Because patients with OAG and myopia have characteristic optic disc deformation [23,24,25] and a higher prevalence of central visual impairment [12,20,21], the present study assessed whether optic disc morphology, as determined using the optic disc tilt ratio, was associated with the occurrence of paracentral scotoma. This study found that a higher tilt ratio was the only factor significantly associated with the occurrence of paracentral scotoma in Korean patients with OAG and myopia. Although the optic disc tilt ratio was significantly associated with a nasal location of CRVT, longer AXL, and lower VF mean deviation, in agreement with previous findings [16,26,27], these three factors were not associated with the occurrence of paracentral scotoma. A higher optic disc tilt ratio was previously shown to be associated with an increased risk of parafoveal scotoma in myopic patients with early glaucoma [21]. A longer AXL may indicate a greater degree of posterior scleral deformation, leading to a CRVT shift accompanied by a lamina cribrosa shift and resulting in axonal damage [26]. Axial elongation causing a change in ONH morphology characterized by a clinically detectable optic disc tilt may result in axons in the papillomacular bundle or foveal vulnerability zone being more prone to damage [28]. This hypothesis is supported by findings showing relationships between optic disc tilt, localized papillomacular bundle defects near the fovea, and parafoveal VF impairment [11,12].

The nasalization of CRVT has been associated with central VF defects [22,29]. It has been suggested that CVRT nasalization may reduce mechanical support for the lamina cribrosa in the temporal region, increasing susceptibility to glaucomatous damage [22,29]. Alternatively, it was speculated that the nasalization of CRVT may affect the adequacy of vascular supply in the temporal region, leading to retinal nerve fiber layer (RNFL) thinning in the macular region [22,29]. The present study found, however, that the location of CRVT did not directly affect the occurrence of parafoveal scotoma. The decentration of the CRVT in the ONH results from displacement between the lamina cribrosa and scleral canal opening. Myopic axial elongation is frequently accompanied by temporal shifts of the scleral canal opening and nasal shifts of lamina cribrosa sheets, leading to the nasalization of CRVT within the optic disc in most eyes. The finding that a nasal position of the CRVT was not associated with paracentral scotoma indicates that the CRVT location itself does not necessarily represent the stress on the papillomacular axons in myopic eyes. However, the CVRT location in the present study was based on two-dimensional images, not on three-dimensional structures deep within the ONH. CVRT location varies between individual eyes, with accurate assessment of CRVT location being difficult due to the many paths even within the lamina cribrosa. Therefore, it remains unclear whether CRVT location, as determined using current methodologies, can explain the preferential location of glaucomatous damage in myopic eyes.

Despite the degree of myopia being similar in the three groups of eyes, the tilt ratio was significantly higher in eyes with paracentral scotoma than in eyes with peripheral scotoma or those without VF defects, but did not differ in the latter two groups. Optic disc tilt was previously reported to be unrelated to the presence of VF defects in eyes with myopia and OAG, when the location of VF defect was not considered [19]. These findings, together with the present results, indicate that optic disc tilt itself may not be a risk factor for the development of glaucomatous VF defects, but is a specific risk factor for the development of paracentral scotoma. 

The relationship between optic disc tilt and VF defects remains unclear. Optic disc tilt was not significantly associated with the central dominant VF defect in myopic eyes with normal tension glaucoma [20]. The discrepancy between studies may have been due to methodological differences in defining central VF defects or in differences between study populations. The previous study used SITA 10-2 and SITA 24-2 to assess central and peripheral visual function, respectively, and compared the MD of the two tests to determine which VF defect is dominant. In addition, the previous study included eyes with normal tension glaucoma, in which decreased ocular perfusion likely played an important role in glaucoma pathogenesis, along with mechanical stress. Moreover, parafoveal scotoma may be an indicator of decreased perfusion [30,31,32,33]. 

There are some limitations to the present study. First, groups were classified based on the presence or absence of paracentral scotoma; thus, some in the paracentral scotoma group had diffuse VF defects involving both central and peripheral VF. It would have been ideal to include eyes having earlier VF damage with only paracentral or peripheral scotoma. However, such cases were limited in our database of OAG and myopia. Reducing the sample size may have reduced the statistical power of these analyses. This limitation was unlikely to have affected the main results of this study, as VF severity did not differ between the paracentral and peripheral scotoma groups, and all eyes had relatively early to moderate VF damage (average VF mean deviation = −2.80). A second limitation was that this study did not evaluate the axis of optic disc tilt relative to the fovea. Although tilt axis has been associated with the location of hemifield VF defect in glaucoma patients with myopia, the determination of this topographic association was outside of the scope of this study. Third, the optic disc tilt ratio was manually determined; therefore, the optic disc border may have been ambiguous when measuring the diameters of the optic disc, especially in eyes with profound optic disc deformation. The excellent interobserver agreement in the measurement of optic disc tilt suggests, however, that this limitation was unlikely to have resulted in significant error. Fourth, the location of CRVT was based on an arbitrary definition. We assumed that the CRVT location within the nasal one third of disc diameter could be assuredly considered nasal. A significant relationship between the CRVT location and optic disc tilt was found, which may suggest the robustness of our definition of the CRVT location. Finally, systemic factors were not fully considered because of the retrospective design of this study. Although previous studies suggested that systemic factors may be associated with the occurrence of paracentral scotoma [33,34,35,36], the mechanism of central VF damage may differ in glaucomatous eyes with and without myopia. Because the present study was specifically designed to account for central VF damage in myopic eyes of relatively young patients, systemic factors were less likely to be associated with the occurrence of paracentral scotoma.

In conclusion, optic disc tilt in myopic OAG patients was associated with greater odds of paracentral scotoma. These findings may provide insights into the mechanism underlying the association between optic disc tilt and the location of VF damage. Further studies are needed to determine methods of preventing the development and progression of central VF defect in myopic eyes, as well as preventing myopia progression. 

## Figures and Tables

**Table 1:** Comparison of the demographic and clinical characteristics between the myopic OAG eyes with paracentral scotoma, those with peripheral scotoma, and those without visual field defects.

	Paracentral Scotoma A (*n* = 99)	Peripheral Scotoma B (*n* = 65)	No VF Defects C (*n* = 47)	*p*-Value	Post Hoc Test ^†^ (Group)
Age, years	53.6 ± 12.2	51.31 ± 11.6	51.4 ± 11.5	0.389	
Sex (female/male), *n* *	33/66	30/35	18/29	0.256	
Location of CRVT (central/nasal), *n* *	34/65	18/47	19/28	0.364	
Tilt ratio	1.40 ± 0.32	1.31 ± 0.17	1.28 ± 0.22	**0.018**	A > B = C
Baseline IOP, mmHg	16.57 ± 5.00	16.43 ± 4.10	15.83 ± 3.44	0.633	
SEQ, diopters	−4.63 ± 3.57	−4.81 ± 3.15	−5.16 ± 4.76	0.737	
AXL, mm	26.45 ± 1.20	26.37 ± 1.52	26.5 ± 1.19	0.885	
CCT, μm	542.7 ± 41.0	545.4 ± 43.5	529.4 ± 38.9	0.117	
Global RNFLT, μm	72.75 ± 11.32	74.38 ± 10.50	82.26 ± 8.96	**<0.001**	A = B > C
Visual field MD, dB	−3.59 ± 1.90	−3.23 ± 2.17	−0.45 ± 1.26	**<0.001**	A = B > C
Visual field PSD, dB	4.64 ± 2.65	4.21 ± 2.64	1.55 ± 0.71	**<0.001**	A = B > C

* Comparison was based on chi-square test. Other comparisons were based on one-way analysis of variance (ANOVA). ^†^ Tukey’s post hoc test. Statistically significant values are shown in bold. CRVT = central retinal vessel trunk; IOP = intraocular pressure; SEQ = spherical equivalent; AXL = axial length; CCT = central corneal thickness; RNFLT = retinal nerve fiber layer thickness; MD = mean deviation; PSD = pattern standard deviation.

**Table 2:** General linear model to determine the factors associated with optic disc tilt ratio.

	Univariable Analysis			Multivariable Analysis *		
	*β*	95% CI	*p*-Value	*β*	95% CI	*p*-Value
Age, per 1 year older	−0.001	−0.004~0.002	0.357			
Female sex	0.002	−0.073~0.076	0.968			
Nasal location of CRVT	0.201	−0.273~−0.130	**<0.001**	0.212	−0.287~−0.137	**<0.001**
Baseline IOP, per 1 mmHg higher	−0.004	−0.012~0.005	0.387			
SEQ, per 1 diopter larger	−0.009	−0.019~0.001	**0.069**	0.002	−0.008~0.012	0.7
AXL, per 1 mm longer	0.05	0.021~0.079	**0.001**	0.044	0.015~0.072	**0.003**
CCT, per 1 μm thicker	0.00	−0.001~0.001	0.407			
Global RNFLT, per 1 μm thicker	0.001	−0.002~0.005	0.392			
Visual field MD, per 1 dB larger	−0.025	−0.041~−0.009	**0.022**	−0.018	−0.034~−0.003	**0.021**
Visual field PSD, per 1 dB larger	0.008	−0.006~0.022	0.272			

* Only variables with a *p*-value of less than 0.10 in the univariable analysis were included in the multivariable model. Statistically significant values are shown in bold. CI = confidence interval; CRVT = central retinal vessel trunk; IOP = intraocular pressure; SEQ = spherical equivalent; AXL = axial length; CCT = central corneal thickness; RNFLT = retinal nerve fiber layer thickness; MD = mean deviation; PSD = pattern standard deviation.

**Table 3:** Logistic regression analysis to determine the factors associated with the presence of paracentral scotoma.

	Univariable Analysis			Multivariable Analysis *		
	OR	95% CI	*p*-Value	OR	95% CI	*p*-Value
Age, per 1 year older	1.016	0.990~1.043	0.235			
Female sex	0.583	0.307~1.109	0.100			
Nasal location of CRVT	1.366	0.689~2.706	0.371	0.488	0.216~1.103	0.085
Optic disc tilt ratio	4.498	1.043~19.397	**0.044**	**8.832**	**1.369~56.960**	**0.022**
Baseline IOP, per 1 mmHg higher	1.007	0.940~1.078	0.85			
SEQ, per 1 diopter larger	1.015	0.926~1.113	0.746			
AXL, per 1 mm longer	1.048	0.814~1.349	0.718	0.938	0.710~1.240	0.652
CCT, per 1 μm thicker	0.998	0.991~1.006	0.696			
Global RNFLT, per 1 μm thicker	0.986	0.959~1.015	0.351			
Visual field MD, per 1 dB larger	0.912	0.777~1.070	0.259	0.936	0.788~1.113	0.457
Visual field PSD, per 1 dB larger	1.067	0.944~1.206	0.302			

This analysis included only perimetric glaucoma (*n* = 164). * Variables that were significantly associated with optic disc tilt ratio were considered confounding factors and were included in the multivariable model. Statistically significant values are shown in bold. OR = odds ratio; CI = confidence interval; CRVT = central retinal vessel trunk; IOP = intraocular pressure; SEQ = spherical equivalent; AXL = axial length; CCT = central corneal thickness; RNFLT = retinal nerve fiber layer thickness; MD = mean deviation; PSD = pattern standard deviation.

## Data Availability

The data presented in this study are available on request from the corresponding author. The data are not publicly available due to privacy regulations.
